# 3D-Printed, Dual Crosslinked and Sterile Aerogel Scaffolds for Bone Tissue Engineering

**DOI:** 10.3390/polym14061211

**Published:** 2022-03-17

**Authors:** Ana Iglesias-Mejuto, Carlos A. García-González

**Affiliations:** I+D Farma Group (GI-1645), Department of Pharmacology, Pharmacy and Pharmaceutical Technology, Faculty of Pharmacy, iMATUS and Health Research Institute of Santiago de Compostela (IDIS), Universidade de Santiago de Compostela, E-15782 Santiago de Compostela, Spain; ana.iglesias.mejuto@rai.usc.es

**Keywords:** 3D-printing, glutaraldehyde, aerogel, hydroxyapatite, bone scaffold

## Abstract

The fabrication of bioactive three-dimensional (3D) hydrogel scaffolds from biocompatible materials with a complex inner structure (mesoporous and macroporous) and highly interconnected porosity is crucial for bone tissue engineering (BTE). 3D-printing technology combined with aerogel processing allows the fabrication of functional nanostructured scaffolds from polysaccharides for BTE with personalized geometry, porosity and composition. However, these aerogels are usually fragile, with fast biodegradation rates in biological aqueous fluids, and they lack the sterility required for clinical practice. In this work, reinforced alginate-hydroxyapatite (HA) aerogel scaffolds for BTE applications were obtained by a dual strategy that combines extrusion-based 3D-printing and supercritical CO_2_ gel drying with an extra crosslinking step. Gel ageing in CaCl_2_ solutions and glutaraldehyde (GA) chemical crosslinking of aerogels were performed as intermediate and post-processing reinforcement strategies to achieve highly crosslinked aerogel scaffolds. Nitrogen adsorption–desorption (BET) and SEM analyses were performed to assess the textural parameters of the resulting alginate-HA aerogel scaffolds. The biological evaluation of the aerogel scaffolds was performed regarding cell viability, hemolytic activity and bioactivity for BTE. The impact of scCO_2_-based post-sterilization treatment on scaffold properties was also assessed. The obtained aerogels were dual porous, bio- and hemocompatible, as well as endowed with high bioactivity that is dependent on the HA content. This work is a step forward towards the optimization of the physicochemical performance of advanced biomaterials and their sterilization.

## 1. Introduction

Large bone defects caused due to osteosarcoma interventions or traumatic fractures can be regenerated using bone grafts to promote spontaneous biological bone healing processes [1]. Standard clinical practices in bone defect repair include autografts, vascularized autografting, allografts and xenografts. Nevertheless, these alternatives have important difficulties to consider with regard to the formation of blood vessels, short shelf lives and high costs [2]. Bone tissue engineering (BTE) aims to regenerate bone tissue at the host defect site in an environment mimicking the natural bone extracellular matrix (ECM). Bone graft composition governs their biocompatibility, whereas their porous structure rules cell penetration and nutrient diffusion. Namely, biodegradable scaffolds for BTE promote bone formation and should eventually degrade in situ at a rate compatible with the pace of new tissue formation [2].

An ideal bone scaffold should mimic the ECM while providing suitable anatomical and functional properties to the damaged area in order to preserve its physiological role [3]. Alginate scaffolds swell easily, allowing cell penetration inside the matrix scaffold [4]. Additionally, alginate is a biocompatible and hydrophilic copolymer of β-L-guluronic acid and α-D-mannuronic acid units, which is able to form stable gels in the presence of divalent cations, such as Ca^2+^ [5]. However, alginate scaffolds present limitations for bone defect treatment due to their poor bioactivity [6]. Accordingly, hydroxyapatite (HA) is used as filler in BTE composite scaffolds because of its high biocompatibility, strong reabsorption ability, adequate cell adhesiveness and outstanding bioactivity [7,8].

Alginate bioinks are widely used to obtain three-dimensional (3D) scaffolds through different 3D-printing technologies because of their low toxicity and easy/fast crosslinking ability. 3D-printing is a disruptive and precise technology for the processing of BTE scaffolds with patient-specific shapes using a computer-aided design (CAD) [9,10]. 3D- printing is used to deposit living and non-living materials in a predesigned 2D or 3D CAD pattern using layer-by-layer manner to fabricate bioengineered structures for regenerative medicine applications [11]. Bioprinting methods for BTE can be classified by the technology used in three groups: light-based, material jetting (inkjet, microvalve, laser and acoustic printing) and material extrusion [12,13]. Light-based printing by stereolithography, digital light processing or two-photon polymerization exploits the use of a light pulse onto a bioink to induce its photopolymerization in a spatially selective manner and in repeated cycles to obtain complex 3D designs. This technology is fast and has a high resolution but uses complex equipment in terms of process control. Additionally, UV-light and light-emitting diodes can induce cell damage and ink degradation. Material jetting or drop-on-demand (DOD) printing is a technology able to obtain 2D and 3D scaffolds by material patterning dropwise (nano to picoliter droplet range) and allows us to incorporate cells into personalized designs with high resolutions. Cell patterning by DOD printing allows stepping in highly advanced tissue engineering grafts; however, the inks should have low viscosity for jetting, and their application is usually limited to soft tissue engineering. On the other hand, continuous or extrusion-based printing is a particularly promising alternative for the fabrication of scaffolds to regenerate human tissues [14]. Extrusion-based printing is an automated manufacturing technique that allows biomaterial deposition with highly complex 3D structures to create a substrate through a pneumatic- or mechanical-driven extrusion. This method can use inks with high viscosities and is easily scalable, reproducible, and results in personalized scaffolds to allow the fabrication of patient-specific tissue-engineered constructs [15]. Precise control of the nanoscale topography of 3D-printed scaffolds is still a technological challenge usually carried out by post-processing approaches (e.g., calcium phosphate coating) [16,17,18].

Aerogels are advanced nanostructured materials with high mesoporosity, among other extraordinary and tunable physicochemical properties (high specific surface area with low density) of interest for regenerative medicine [19,20,21,22]. Aerogels are typically obtained by the supercritical (sc-)drying of gels, as this solvent extraction approach can preserve the original hydrogel nanostructure in the dry form. For this reason, the aerogel technology could help to solve the lack of nanostructuration of the as-printed structures, one of the current 3D-printing limitations [23]. Nevertheless, the production of aerogels with customized external and internal structures is still a remarkable challenge [24,25]. In this context, the technological combination of 3D-printing technology and supercritical drying results in alginate-HA aerogel-based scaffolds with a high fidelity with respect to the original CAD design and the complex nanostructure required for BTE applications [7]. Nevertheless, these 3D-printed aerogels should have enhanced properties regarding their degradation rate behavior in biological fluids, their mechanical integrity and their sterility conditions to be compliant with regulations for implantable medical devices.

BTE grafts substitutes should be manufactured as a structure that mimics the biomechanical behavior of native bone [6,26] and bears the anatomical and functional efforts that the damaged area suffers [27,28]. Different alternatives are employed to improve the performance of the scaffolds aimed at BTE [29,30]. For instance, HA is commonly employed to yield biological and structural properties comparable to that of human bone [26,31]. Additionally, crosslinking strategies enhance the mechanical, biological and degradation properties of biomaterials such as bone substitutes [32,33]. This process of network bonding formation is essential in scaffolding to improve the biomechanical performance of implants [34,35]. Chemical crosslinking is more effective than physical crosslinking (e.g., UV-irradiation, dehydrothermal treatment) and is extensively used to manufacture bone substitutes due to its ability to undergo chemical modifications in the polymeric backbone that yields high crosslinking degrees [34]. Glutaraldehyde (GA) is a commonly employed chemical crosslinker on biopolymeric scaffolds [36,37] that significantly improves the reinforcement and durability of biomaterials [33,38]. GA reacts with amine or hydroxyl groups of polymers through a Schiff-base reaction that results in strongly crosslinked networks [39]. Nevertheless, aldehyde groups of unreacted GA are toxic for cells as they induce inflammation [40,41]. Thus, several detoxifying strategies are developed to increase the biocompatibility and durability of GA-crosslinked scaffolds [42], such as washing the structures crosslinked with GA with free amine groups solutions or amino acid solutions to remove free aldehyde groups [33]. On the other hand, the combination of different crosslinking methods to reduce cytotoxic effects and to improve the cost-effectiveness of the overall process is nowadays a trend in tissue engineering [33,43].

Finally, the sterilization of biomaterials prior to being implanted is crucial to avoid post-surgical complications [44]. Sterilization is defined as the absence of life and biological agents (fungi, bacteria and viruses) [45]. It is commonly achieved by heat, chemicals, irradiation, pressure or filtration by membranes. Common sterilization techniques such as steam, ethylene oxide and gamma (γ) irradiation impact negatively on scaffold structures affecting their physicochemical properties and, consequently, their biological performance [46]. The use of supercritical CO_2_ (scCO_2_) in combination with chemical additives (at ppm contents) is a low-temperature radiation-free alternative to sterilize biomaterials [47].

The recently proposed combination of 3D-printing and supercritical CO_2_ technology yielded BTE scaffolds with high biological and structural performance [7]. A dual crosslinking strategy (CaCl_2_ ageing and GA vapor) is herein proposed with the aim of improving the aerogel’s integrity and customization while maintaining its superior performance and fidelity to the original CAD file. In this work, extrusion-based 3D-printing technology will be employed to obtain personalized hydrogel-based scaffolds with a complex nanostructure preserved after supercritical CO_2_ drying step to finally obtain 3D-aerogel-based scaffolds with structural integrity and customized designs. The dual crosslinking of alginate-HA aerogel scaffolds for BTE aimed at personalized medicine will be obtained by the CaCl_2_ gelation and ageing of hydrogels, as well as by GA-crosslinking on the aerogel structures. Bio- and hemocompatibility, bioactivity and textural properties of the end structures will be evaluated regarding personalized medicine and BTE applications. Finally, scCO_2_ is also herein evaluated as a sterilizing technique to obtain personalized aerogel scaffolds ready for patient implantation.

## 2. Materials and Methods

### 2.1. Materials

Alginic acid sodium salt from brown algae with medium viscosity (guluronic acid/mannuronic acid ratio: 70/30, *M*w 403 kDa, 3170 cps) and calcium chloride (CaCl_2_; *M*w 110.98 g/mol, 99.99% purity) were provided by Sigma Aldrich (Madrid, Spain). Hydroxyapatite (HA; *M*w 502.31 g/mol, reagent grade purity, micropowder) was provided by Fluidinova (Moreira da Maia, Portugal), glutaraldehyde (GA; 50% aqueous solution) by Scharlau (Barcelona, Spain) and hydrogen peroxide (H_2_O_2_; 30% aqueous solution) by Honeywell Fluka (Madrid, Spain). CO_2_ (purity > 99.9%) was supplied by Nippon Gases (Madrid, Spain) and absolute ethanol (EtOH) by VWR (Radnor, PA, USA). Water was purified using reverse osmosis (resistivity > 18 MΩ·cm; Milli-Q, Millipore^®^, Madrid, Spain).

### 2.2. 3D-Printing of Hydrogel Scaffolds

Alginate inks were prepared using Milli-Q water as a solvent. Different HA concentrations (0, 8, 16 and 24 wt.% with respect to water) were added to 6 wt.% alginate solution. Inks were prepared under vigorous agitation (600 rpm), employing a homogenizer (VWR vos 60, Radnor, PA, USA) for at least 1 h at room temperature (RT). The obtained alginate solutions were degassed in a sonication bath (Branson 3510 Emerson, Ferguson, MO, USA) for 10 min to eliminate air bubbles. Hydrogels were obtained via the 3D-printing of the alginate inks with a Cellink BIOX Bioprinter (Boston, MA, USA) at room temperature, using an extrusion printhead with a 3-mL syringe and a 410-µm nozzle at the printing pressure of 60 kPa, and a printing velocity of 12 mm/s. Scaffolds with dimensions of 20 × 20 × 1 mm were obtained with a grid pattern and three layers. After the printing process, all scaffolds were put in contact with 1 M CaCl_2_ aqueous bath solutions for gelation during 1 h (ageing step). For the comparison of the physicochemical characterization, gel scaffolds of the same formulations were gelled in 1 M CaCl_2_ solution without the ageing step, and others were put in contact with 0.5 M CaCl_2_ bath solutions aged for 1 h.

### 2.3. Rheological Evaluation of Alginate-HA Inks

Alginate-HA inks were mechanically characterized by applying two independent tests at 20 °C. Flow behavior was recorded between 0.05 and 200 rad/s in a Rheolyst AR-1000N rheometer (TA Instruments, Newcastle, UK) equipped with a Peltier plate and a cone geometry (40 mm diameter, 2°) with a solvent trap. Viscoelastic behavior and shear-thinning properties were analyzed using a rheometer (Anton Paar MCR 302, Graz, Austria) fitted with a H PTD 200 Peltier hood and a disposable measuring aluminum plate (15 mm diameter) with 1 mm gap. Five steps in amplitude sweep mode were performed to simulate the rests and high-stress conditions of the inks before, during and after printing. Thus, the storage (G’) and the loss (G”) moduli were recorded at (i) 0.5% shear strain at 1 Hz for 300 s (data points every 12 s); (ii) 100% shear strain at 1 Hz for 120 s; (iii) 0.5% shear strain at 1 Hz for 300 s; (iv) 100% shear strain at 1 Hz for 120 s; and (v) 0.5% shear strain at 1 Hz for 300 s [48,49].

### 2.4. Supercritical Drying of 3D-Printed Gels

Prior to drying, alginate hydrogels were immersed in absolute ethanol after ionic ageing in 1 M CaCl_2_ for 1 h. Two solvent exchanges to ethanol were carried out with an exchange frequency of 24 h. The alcogels were then wrapped in filter paper cartridges and dried by supercritical drying to obtain the aerogels. Briefly, gels were placed into a 100-mL stainless steel autoclave (Thar Process, Pittsburg, PA, USA) and immersed in 25 mL of absolute ethanol. scCO_2_ was supplied using a dual-piston pump and introduced from the top of a vessel heated at the constant temperature of 40 °C. Firstly, a continuous CO_2_ flow rate (5–7 g/min) at 120 bar took place for 4 h. Aerogels were then collected from the autoclave after a CO_2_ depressurization rate of 2 bar/min and stored for characterization. The obtained scaffolds were denoted as Alg 6 wt.%, HA y%, where y denotes the HA content (0, 8, 16, 24 wt.%). The suffix CaCl_2_ 1M1h (alternatively CaCl_2_ 0.5M1h) denotes aged aerogels, while CaCl_2_ 1 M denotes non-aged aerogels.

### 2.5. Aerogel Post-Crosslinking with Glutaraldehyde Vapor

Certain aerogels were crosslinked in a GA-saturated atmosphere for 20 min by placing the aerogels inside a desiccator under ambient pressure and temperature conditions and containing a 25% (*v*/*v*) GA aqueous solution (100 mL) at the bottom [50] and without direct physical contact with the aerogels. The aerogels were maintained for 1 h in the GA atmosphere, and a vacuum was then applied for 20 min to remove the unreacted glutaraldehyde. The obtained GA-crosslinked scaffolds were denoted by the suffix −GA (Table 1).

### 2.6. Physicochemical Characterization of Alginate Aerogel Scaffolds

The skeletal density of the aerogels (*ρ*_skel_) was determined using a helium pycnometer (Quantachrome, Boynton Beach, FL, USA) at room temperature (25 °C) and pressure of 1.01 bar. Values were obtained from five replicates. Envelope density (*ρ*_env_) was measured using the GeoPyc 1360 Envelope Density Analyzer (Micromeritics, Norcross, GA, USA), employing DryFlo^®^ powder as the fluid medium. The applied force was 25 N, and each sample was measured in five cycles. The standard deviation of the measured values was 0.002 g/cm^3^. Scaffold porosity (*ε*) was determined according to Equation (1):

*ε* (*%*) = [1 − (*ρ*_env_/*ρ*_skel_)] × 100 (1)

The volume shrinkage (in percentage) of the dual crosslinked aerogel scaffolds was calculated from the external dimensions of the structures before supercritical drying and after performing the dual crosslinking strategy following Equation (2):

Volume shrinkage (%) = [(Alcogel volume − Aerogel volume)/Alcogel volume] × 100 (2)

Low-temperature N_2_ adsorption–desorption analysis (ASAP 2000 Micromeritics Inc.; Norcross, GA, USA) was performed to assess aerogel textural properties. Before measurements, aerogels were degassed under vacuum at 40 °C for 24 h. The specific surface area (A_BET_) of the aerogels was evaluated employing the BET (Brunauer–Emmett–Teller) method. Specific pore volume (V_p_), pore size distribution and mean pore diameter (d_p_) were determined applying the BJH (Barrett–Joyner–Halenda) method. The morphology of the aerogels was evaluated by scanning electron microscopy (SEM, EVO LS15, Zeiss, Oberkochen, Germany). Aerogel samples were iridium-sputtered prior to imaging to minimize charging and to improve the image quality. Finally, Attenuated Total Reflectance/Fourier-Transform Infrared Spectroscopy (ATR/FT-IR) was performed with a Gladi-ATR accessory using a diamond crystal (Pike, Madison, WI, USA) to evaluate the chemical structure of the dual crosslinked aerogel scaffolds. Raw sodium alginate and alginate aerogel scaffolds were characterized in the mid-IR spectrum range (400–4000 cm^−1^) using 32 scans and at a resolution of 2 cm^−1^.

### 2.7. Evaluation of the Bioactivity of the Alginate-HA Aerogel Scaffolds

An in vitro test using simulated body fluid (SBF) was used to assess the bioactivity of the aerogels [51]. The SBF solution was prepared with ion concentrations nearly equal to those of human blood plasma. To perform the bioactivity tests, scaffolds were cut into pieces of 1 × 1 × 0.1 cm dimensions and immersed in 40 mL of SBF pH 7.45 solution in plastic conical tubes at 37 °C for 3, 7, 15 and 28 days. At the end of the experiment, the wet gels were immediately rinsed with deionized water and dried by lyophilization. Bioactivity was analyzed by SEM focusing on the surface crystals after the mineralization process and by EDX analysis [52].

### 2.8. Cell Viability Tests

The cytocompatibility of the different alginate-HA aerogel scaffolds was determined by assessing the viability of mouse embryo fibroblasts (BALB/c 3T3) after 24 and 48 h of culture in the presence of the aerogel formulations using the WST-1 test and in triplicate. This test is based on the degradation of WST-1 into formazan and is directly correlated with the number of metabolically active cells. BALB cells (6500 cells/cm^2^) were seeded in 24-well plates in DMEM supplemented with 15% fetal bovine serum, penicillin 100 U/mL and streptomycin 100 g/mL. Cells were incubated at 37 °C in a humidified atmosphere enriched with 5% CO_2_. Scaffolds (1 × 1 × 0.1 cm) were UV-sterilized for 1 h and then placed in the wells with cells containing 1500 µL of DMEM supplement. Positive controls of cells with 1000 µL of medium and blanks of 1000 µL of medium (both in triplicate) were maintained at the same conditions. After 24 and 48 h of culture, scaffolds were removed, 250 µL of the medium was left in the wells and 25 µL of WST-1 reagent was added. The plate was incubated for 2 h at the same conditions and then shaken thoroughly for 1 min. Finally, 110 µL were transferred to a 96-well plate to measure the absorbance at λ = 450 nm in a microplate reader (Infinite^®^ M200, Tecan Group Ltd., Männedorf, Switzerland).

### 2.9. Hemolytic Activity Test

The hemolytic activity of the alginate-HA aerogel scaffolds was tested using human blood (Galician Transfusion Center, Spain) obtained in accordance with the rules of the Declaration of Helsinki. Fresh human whole blood was diluted to 33% (*v*/*v*) in 0.9% (*w*/*v*) NaCl solution and 1 mL of the diluted blood was transferred to Eppendorf tubes containing either 1 × 1 × 0.1 cm aerogel scaffolds, 100 μL of 4% (*v*/*v*) Triton X-100 (positive control) or 100 μL of 0.9% (*w*/*v*) NaCl (negative control). Aerogels were incubated for 60 min at 37 °C and 100 rpm in an orbital shaker and then centrifuged at 10,000× *g* for 10 min (Sigma 2-16P, Sigma Laboratory Centrifuges, Osterode am Harz, Germany). Finally, 150 μL of the supernatant were transferred to a 96-well plate, and the absorbance of the hemoglobin was measured at λ = 540 nm (FLUOStar Optima, BMG Labtech, Ortenberg, Germany). The percentage of hemolysis of the aerogels was determined using Equation (3):

Hemolysis (%) = (Abs_s_ − Abs_n_)/(Abs_p_ − Abs_n_) × 100 (3)

where Abs_s_ is the absorbance of the samples containing the aerogels, Abs_n_ is the absorbance of the negative control (0% of hemolysis), and Abs_p_ is the absorbance of the positive control (100% of hemolysis). All tests were carried out in triplicate.

### 2.10. Supercritical Sterilization of Dual Crosslinked Aerogels

Dual crosslinked aerogel scaffolds were introduced into individually and thermally sealed sterilization pouches. Then, they were placed in a high-pressure 600-mL autoclave (NovaGenesis, NovaSterilis Inc., Ithaca, NY, USA) with 1200 ppm of H_2_O_2_, which was heated until 40 °C and pressurized with CO_2_ until 100 bar. After 30 min contact time, the system was depressurized at a constant flow rate of 5 bar/min until atmospheric pressure. Aged and dual crosslinked alginate-HA formulations were then seeded in tryptic soy agar (TSA) plates for colony forming units (CFU) quantification and also incubated for 24 h in trypto-casein soy broth (TSB). The structural stability of sterilized aerogels was evaluated in terms of textural properties by BET and SEM analyses.

### 2.11. Statistical Analysis

Results of cell viability tests for each aerogel scaffold type (*n* = 3) were reported as mean value ± standard deviation. *t*-tests were carried out to determine the statistical significance of the differences among the groups, and values of *p* < 0.05 were considered as statistically significant.

## 3. Results and Discussion

### 3.1. Rheological Properties of Alginate-HA Inks

**Figure 1:**
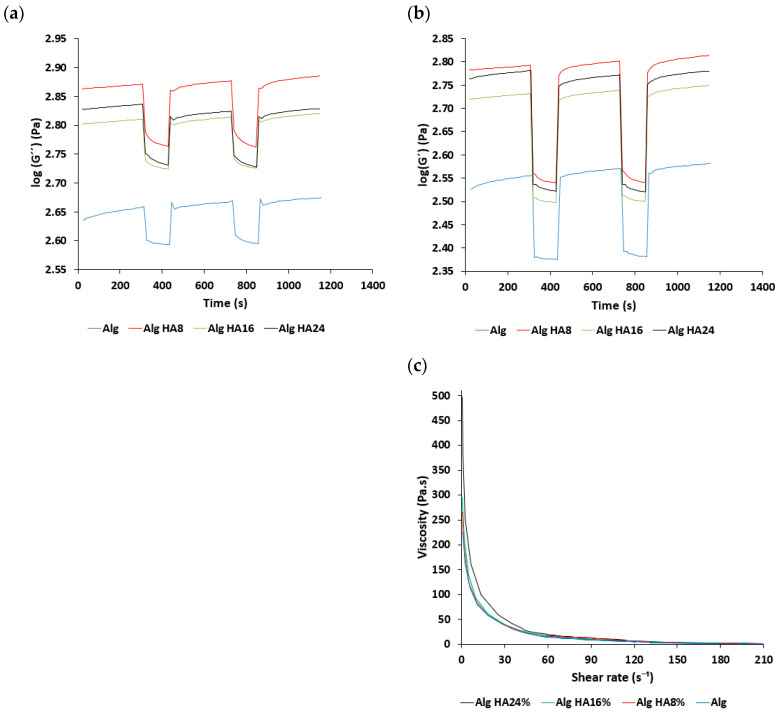
Rheological characterization of alginate-HA inks: (**a**,**b**) Time variation of storage and loss moduli and (**c**) overall viscosity variation with respect to the shear rate for different alginate-HA ink formulations.

Inks for 3D-printed hydrogels should present good biocompatibility, adequate rheological behavior and a quick crosslinking capacity [53]. Specifically, viscoelastic response during the extrusion-based printing process is critical. Under rest-like conditions, G” was higher than G’ for all ink formulations studied (Figure 1a,b), indicating a dominant viscous component. This “liquid-like” behavior is ideal for the 3D-printing extrusion method [54]. The moduli were notably higher for inks containing HA, which evidenced the reinforcement role of this inorganic material in the mechanical properties of the inks. Under high shear strain conditions, both moduli underwent a rapid decrease, which was highly remarkable for G’. This steep decrease in G’, more intense than for G”, suggested that the physical entanglements among the alginate chains easily decreased and the chains oriented in the direction of the stress, which may facilitate the extrusion process. Remarkably, once the strain disappeared, the ink recovered the initial G’ and G” showed at rest, which indicated a rapid recovery of the initial entanglements: This behavior is needed for the ink to maintain the desired shape after extrusion. Sequential cycles of low–high strain evidenced the reproducible mechanical performance of the alginate inks. Flow tests (Figure 1c) confirmed the intense shear-thinning behavior of all inks (with and without HA) at low shear stress conditions, which should facilitate the extrusion process at moderate pressures [55].

### 3.2. Effect of Ageing of Aerogel-Based Scaffolds

The technological combination of 3D-printing and aerogel processing previously used for the preparation of non-aged alginate-HA aerogels [7] was herein coupled with extra crosslinking steps (ageing in a CaCl_2_ aqueous solution and exposing to GA vapor) to manufacture structures with personalized and enhanced crosslinking degrees. The 3D-printing step allows the customization of the scaffolds while the sc-drying processing results in nanostructured aerogels, thus fulfilling two of the requirements for BTE applications.

**Figure 2:**
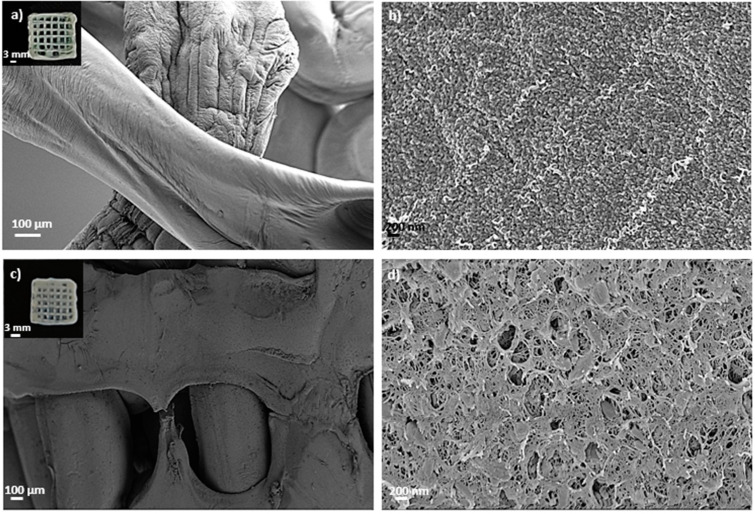
SEM images of 3D-printed aerogel scaffolds prepared from alginate 6 wt.% inks (**a**,**b**) without and (**c**,**d**) with a one-hour ageing step in 1 M CaCl_2_ solution. Aerogels were observed at two different magnifications. Intake in (**a**,**c**): visual appearance of the aerogel scaffolds with a well-defined 3D-pattern.

**Figure 3:**
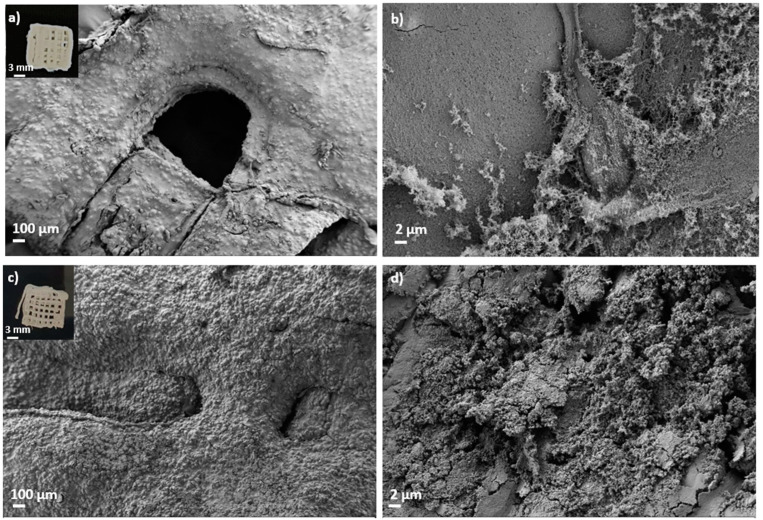
SEM images of 3D-printed and aged aerogel scaffolds prepared from alginate 6 wt.% and HA 16 wt.% inks (**c**,**d**) with and (**a**,**b**) without GA post-crosslinking step. All formulations were crosslinked with GA vapor. Aerogels were observed at two different magnifications. Intake in (**a**,**c**): visual appearance of the aerogel scaffolds with a well-defined 3D-pattern.

Aerogel scaffolds showed, in all cases, homogeneous structures following good shape fidelity with respect to the grid pattern of the CAD design initially printed and composed of fibers with similar dimensions correctly arranged in layers (Figure 2 and Figure 3). In the absence of HA, the hydrogel ageing in 1 M CaCl_2_ gelation bath for 1 h (Alg 6%, HA 0%, CaCl_2_ 1M1h, Table 2 and Figure 2c,d) incremented the textural properties (A_BET_, d_p_ and V_p_) of the resulting aerogels with respect to the same formulations obtained without the ageing step (Alg 6%, HA 0%, CaCl_2_ 1 M, Table 2 and Figure 2a,b). This increase in textural parameters of aerogels was also observed when the one-hour ageing step was alternatively performed in 0.5M CaCl_2_ solution bath (Alg 6%, HA 0%, CaCl_2_ 0.5M1h formulation, Table 2) with respect to the non-aged aerogel counterpart (Alg 6%, HA 0%, CaCl_2_ 0.5 M, Table 2). The same trend in the textural properties between aged and non-aged aerogels was observed in all the cases studied with different HA contents. The ageing step during the aerogel processing increases the crosslinking degree in the end structures (without layers exfoliation and with high structural integrity). This feature, as well as the porous structure with different pore sizes and morphologies observed in SEM images, are highly desirable in end structures for BTE applications.

The incorporation of HA favored the printability of the ink with respect to the ones without HA. Nevertheless, the printability decreased when the HA concentration in the inks reached the threshold value of 24 wt.%, mainly due to the difficulty of the ink extrusion by the printer. This could be due to the increase in strength of alginate hydrogels when increasing HA concentrations are used [56]. It can also be attributed to the chemical reaction between the Ca^2+^ of the HA surface and the -COO^−^ groups of the alginate that results in an ionic bonding that increase the strength of the structures [56]. All the resulting HA-containing aerogel formulations were highly porous structures with mesopores and macropores homogeneously distributed (Figure 3). The highest porosity values (86–87%) were obtained for aerogels without HA and decreased with HA content (Table 2), which is coherent with the density values (*ρ*_skel_ and *ρ*_env_) and with previously reported results for non-aged aerogel formulations [7].

Finally, several macropores are clearly recognized in aged aerogels, which are homogeneously distributed throughout the structure following the CAD design (Figure 2). Macropores in the 100–400 μm range, as in these structures, are considered sufficient to allow bone ingrowth and regeneration [57]. Both biological processes are strongly dependent on the microporosity pattern of the structure as only pore diameters higher than 50 μm promote cell infiltration and bone mineralization [3]. Nevertheless, this pore size range may be detrimental to the scaffold textural properties increasing permeability and degradation [57]. Thus, microporosity and textural parameters (A_BET_, d_p_ and V_p_) must be balanced to promote an appropriate bone tissue regeneration. The enhanced dual porous structure and integrity obtained after the CaCl_2_ ageing step could generate scaffolds that enable the balance between the natural bone healing process and the degradation of the 3D-printed construct.

### 3.3. Effect of GA Chemical Post-Crosslinking of Aerogel-Based Scaffolds

Dual crosslinked aerogel scaffolds obtained after performing the GA vapor crosslinking step exhibit a compact structure with all layers well organized and bonded, resulting in entire, highly crosslinked and personalized structures (Figure 3). The customized 3D-printed pattern was successfully preserved after GA crosslinking as well as the individual fibers and the end scaffold CAD design (Figure 3). SEM images show that the porous structure and pore architecture, as well as the HA granules integrity, were preserved in the aerogel scaffolds after all processing steps (Figure 3c,d). These observations are coherent with previous studies where GA vapor treatment for less than 6 h caused very slight modifications on scaffold architectures, while longer periods induced important modifications on scaffold nano- and microarchitecture and toxicity [39]. Furthermore, the absence of exfoliation layers in the dual crosslinked samples with respect to the non-aged ones induces higher structural integrity in the end aerogel scaffolds that improves the customization of the structures as well as the fulfillment of the BTE requirements. An increase in *ρ*_env_ and a decrease in *ρ*_skel_ was observed after the GA treatment, suggesting higher crosslinking and a more compact end structure in the dual crosslinked aerogels than in the non-GA treated aerogels. Density reduction after GA crosslinking was previously reported in scaffolds with a similar composition for bone regeneration purposes [38]. In general, a decrease in textural properties (A_BET_, V_p_ and *ε*) was observed for all dual crosslinked aerogel formulations with respect to the ones crosslinked only with Ca^2+^ ions (Table 2). Textural properties of the non-aged and GA-crosslinked aerogels (Alg 6%, CaCl_2_ 1 M, GA; and Alg 6%, HA 8%, CaCl_2_ 1 M, GA formulations, Table 2) were also assessed to check if each crosslinking method is contributing, by itself, to the overall decrease in these properties. A similar reduction in textural parameters was found when non-aged and GA-crosslinked aerogels were compared with the non-aged aerogels [7]. A similar trend was previously described in structures with similar composition and crosslinked with GA vapor and/or CaCl_2_ [36,58]. According to SEM images, the decrease in textural parameters could be due to the effect of the crosslinking agent on the morphology of the fibers that leads to the fusion of alginate fibers at their junctions [58].

In general, the dual crosslinking procedure favors the ionic interactions between alginate and Ca^2+^ and the covalent bonds due to GA. These chemical reactions result in aerogel scaffolds with improved integrity, durability and customization with respect to the structures obtained without dual crosslinking. All these features are highly desirable for personalized bone medicine and turn the dual crosslinked alginate-HA aerogel scaffolds into promising candidates for this challenging application.

**Figure 4:**
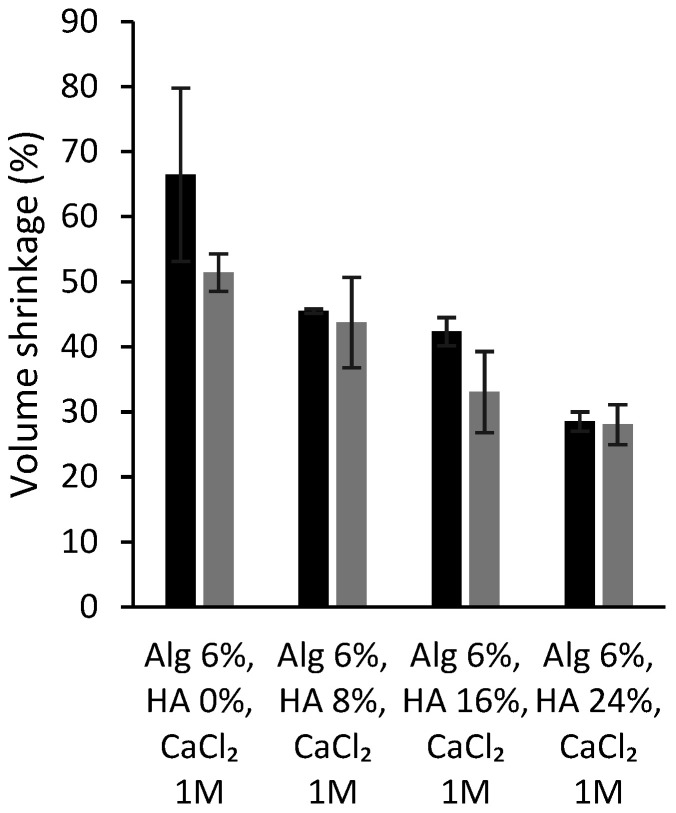
Volume shrinkage (in percentage) of different alginate-HA aerogel scaffolds without (grey bars; source: ref. [7]) and with the dual crosslinking treatment (black bars; this work).

Finally, the volume shrinkage of the dual crosslinked aerogel scaffolds was measured to evaluate if both crosslinking strategies, as well as the direct solvent exchange into ethanol, have a relevant effect in terms of volumetric change in the end structures. The formulations with higher HA content have lower volumetric shrinkage than those without HA (<50%, Figure 4), as previously described for the same structures without the dual crosslinking processing [7]. Furthermore, similar values were found for alginate-HA and dual crosslinked formulations, suggesting that the dual crosslinking strategy does not induce a higher volume shrinkage in the resulting aerogels. Other alginate-based structures show higher shrinkage values (70–80%) with the same solvent exchange steps [59]. The obtained results suggest that the dual processing strategy improves the integrity of the material and reduces volume shrinkage for the obtention of customized aerogel structures.

### 3.4. Chemical Analysis of Alginate-HA Aerogel-Based Scaffolds

**Figure 5:**
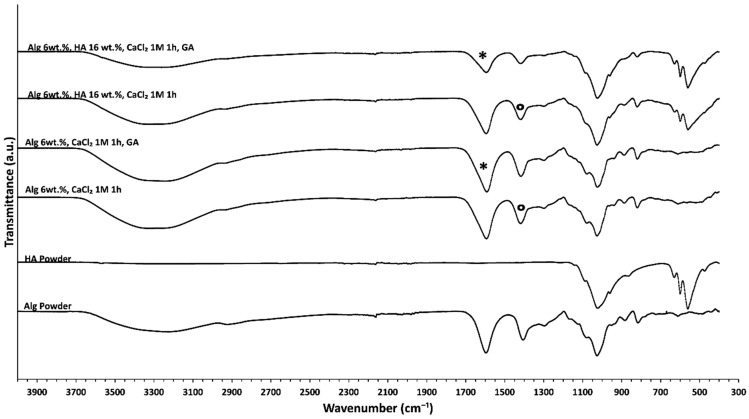
FTIR-ATR spectra of different alginate-HA aerogel formulations. The spectra of the raw materials (HA and Alg) are also included for the sake of comparison.

FTIR spectra of different aerogel scaffolds are displayed in Figure 5. The main differences observed were related to a general reduction in the relative intensity of bands when aerogel formulations are dual crosslinked with respect to the structures aged only with CaCl_2_. This effect is probably due to a weakening in the intramolecular bonding as the bonds stretch to accommodate the coordination structure around the Ca^2+^ ion [60]. Specifically, a decrease in the intensity of the band at 1608 cm^−1^ and the appearance of the band relative to imine at 1644 cm^−1^ suggest effective GA crosslinking (asterisks in Figure 5) [39]. Regarding the GA effect, GA reacts with hydroxyl groups of alginate through a Schiff-base reaction connecting the polymeric chains via intra- or intermolecular bonds and forming a more strongly crosslinked network [61]. Finally, the differences in the pure alginate spectrum with respect to Ca^2+^ crosslinked alginate spectra were due to ionic binding resulting from the interaction between the –COO^−^ groups of alginate with Ca^2+^ as it is shown in all alginate aerogels with respect to alginate powder (Figure 5) [60]. Specifically, the –COO^−^ asymmetric stretching band at 1590 cm^−1^ results from the interaction between Ca^2+^ and alginate –COO^−^ groups (asterisks in Figure 5). Additionally, the –COO^−^ symmetric stretching band at 1414 cm^−1^ is shifted toward lower wavenumbers, thus indicating ionic binding between Ca^2+^ and alginate –COO^−^ (circles in Figure 5) [60].

### 3.5. Bioactivity of Dual Crosslinked Aerogel-Based Scaffolds

Scaffolds can bond to the living bone by forming bone-like apatite on its surface. This in vivo bone bioactivity process can be mimicked in vitro using an SBF medium to predict the apatite formation on the scaffold surface after its immersion in the said medium [62]. Aerogel scaffolds not aged in CaCl_2_ and without post-crosslinking in GA vapor were completely disintegrated and partially dissolved after a short time in an SBF medium (less than 3 days). However, the physical stability of the aerogel scaffolds in aqueous media in the long term was significantly improved after the dual-crosslinking process (i.e., CaCl_2_ ageing and crosslinking with GA vapor). Indeed, GA crosslinking was previously reported to significatively increase the in vitro half-life of collagen matrices [63].

**Figure 6:**
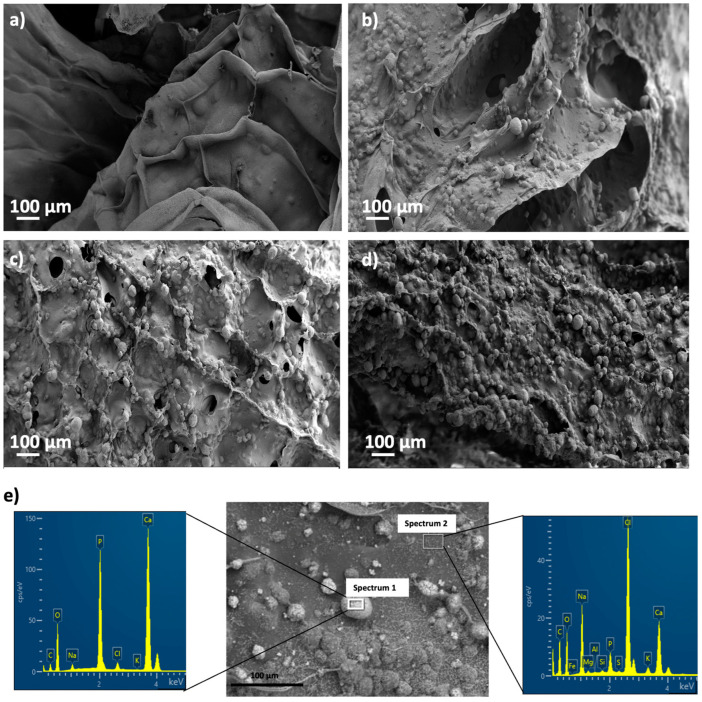
SEM pictures of aerogels printed from alginate 6 wt.% inks and different HA concentrations: (**a**) 0, (**b**) 8, (**c**) 16, and (**d**) 24 wt.%, after bioactivity tests. Aerogels were aged and crosslinked with GA vapor prior to being immersed in SBF medium. (**e**) EDX spectra of Alg 6%, HA 8%, CaCl_2_ 1M1h GA aerogels after SBF treatment in an apatite granule region (Spectrum 1, left) and a background region (Spectrum 2, right), and representative of all the tested samples.

**Figure 7:**
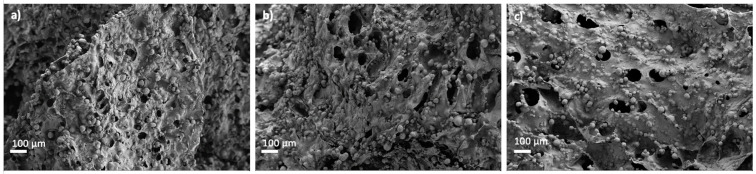
SEM pictures of Alg 6%, HA 24%, CaCl_2_ 1M1h GA aerogels after bioactivity test. Aerogels were left in SBF medium for (**a**) 3, (**b**) 7 and (**c**) 15 days.

White granules appeared on aerogel scaffold surfaces after 28 days of immersion in SBF medium (Figure 6). EDX analysis of these granules confirmed to be calcium phosphate (Figure 6e; spectrum 1) since there was a clear enhancement of the Ca, P and O peaks with respect to the background region (Figure 6e; spectrum 2). This formation of calcium phosphate suggests good binding between organic and inorganic components of the aerogel scaffold, which is essential for the integrity and functionality of the bone [3]. Moreover, a high Ca/P ratio was found after SBF immersion for 28 days, thus suggesting the stimulation of the bone tissue deposition formation and functionality [3,64]. The presence of HA and its uniform distribution throughout the aerogels favored higher bioactivity with increasing HA content (Figure 6), as reported for scaffolds of similar composition (alginate/cellulose/HA) left in SBF for the same time [64]. Finally, HA granules grew during the immersion period in SBF medium at day 28 with respect to the predominant size at day 3 (Figure 7), suggesting a progressive formation of apatite as previously described for alginate-HA and for alginate-bioactive glass composites left in SBF for the same time [65]. The enhancement of the bone mineralization process suggests the potential application of the dual crosslinked alginate-HA aerogel-based scaffolds for BTE.

### 3.6. Biocompatibility Tests for Dual Crosslinked Aerogel-Based Scaffolds

GA was reported to increase the crosslinking density of biomaterials by creating a more rigid network due to the formation of covalent bonds (Schiff bases) [53]. Nevertheless, this application is restricted because the chemical modification generated by GA usually results in significant cytotoxicity, as the aldehyde groups from GA incorporated into biopolymers induce inflammatory responses in soft tissues causing the failure of the regeneration process [41,53].

**Figure 8:**
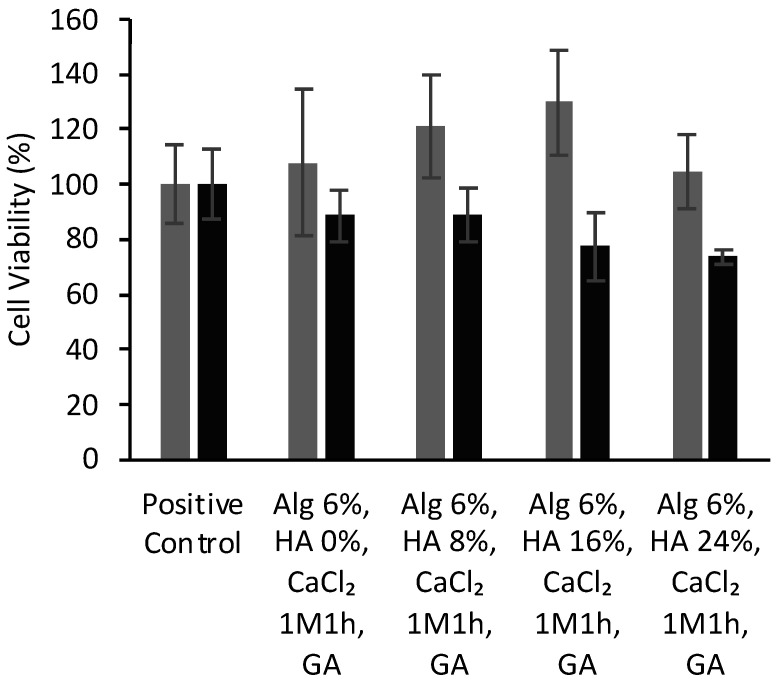
Viability (expressed in %) of BALB cells after 24 (grey bars) and 48 h (black) of contact with alginate-HA-GA aerogel scaffolds determined by WST-1 tests. There were no statistically significant differences among groups (*t*-test; *p* < 0.05).

Figure 8 show the cell viability of BALB cells after 24 and 48 h of culture with different aerogel formulations crosslinked with GA vapor. BALB cells viabilities present values reaching ca. 100% for alginate aerogels, which means that they had no negative effect on cell growth, regardless of HA concentration or GA crosslinking.

The high biocompatibility observed for all the aerogel formulations pointed out that neither the scaffold composition nor the processing strategy compromised the cell viability. The high cell viability observed for these aerogel structures correlates well with scaffolds of similar composition [7,66] and with the biocompatibility of the initial components of the scaffolds (alginate and HA) [67]. Furthermore, the employed strategy avoids the use of chemical agents (i.e., sodium metabisulfite and sodium borohydride) commonly needed after GA treatment to restore the material’s biocompatibility [53]. However, these results contrasted with previous experiments with 3D-structures of similar composition and crosslinked with GA where low cell viability was reported [36]. The different methodology employed to perform the GA-crosslinking process (GA immersion vs. GA atmosphere) may explain these different biological behaviors. These results suggest that the gaseous GA-crosslinking method can shorten the overall processing time with respect to GA-immersion by avoiding the use of detoxifying strategies (e.g., washing with free amine groups solutions or amino acid solutions) commonly needed in the latter case [33,61]. Thus, the GA vapor post-processing method could contribute to the optimization of the GA-based crosslinking to maximize the physicochemical performance of biomaterials and minimize its toxicity risk.

### 3.7. Hemocompatibility Tests for Dual Crosslinked Aerogel-Based Scaffolds

Biomaterials for tissue engineering applications must be cytocompatible with red blood cells and do not interfere with the hemostatic activity since they are going to be placed in a damaged area in direct contact with this cell type. Furthermore, an important stage of the cell proliferation and repair process is the formation of new blood vessels (angiogenesis). Therefore, the determination of the hemocompatibility of the aerogel scaffolds is essential to exclude the possibility of blood traumas that such structures can generate [68]. According to ISO 10993-4 guidelines, materials with hemolysis values lower than 5% can be safely used.

Alginate-HA aerogel scaffolds were highly compatible with the red blood cells with virtually no hemolytic activity (Table 3) and lower hemolytic activity than other HA scaffolds proposed for BTE [69]. GA-crosslinking of aerogels did not alter their hemolytic behavior, so dual crosslinked alginate-HA aerogel-based scaffolds are non-hemolytic biomaterials and meet the requirement for the biological evaluation of medical devices with respect to hemolysis (ISO 10993-4).

### 3.8. Sterilization of Dual Crosslinked Aerogels

**Figure 9:**
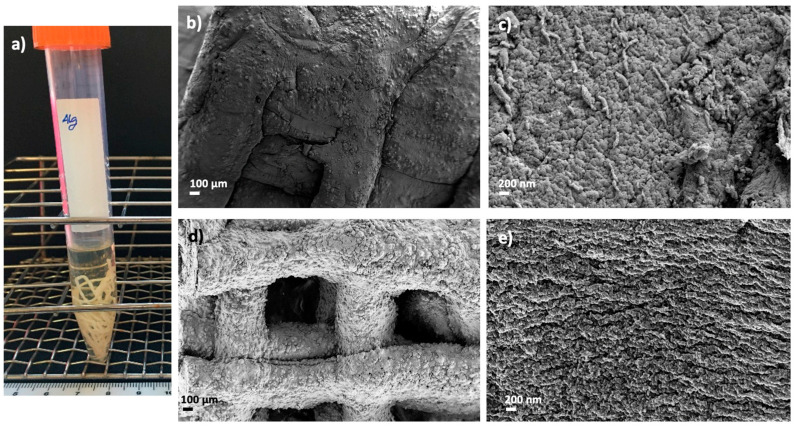
Supercritical sterilization of 3D-printed alginate aerogels: (**a**) Images of sterile and dual crosslinked aerogel scaffolds seeded on TSB tubes after 24 h of culture. Optical and SEM images of sterile (st) and aged aerogel scaffolds: (**b**,**c**) Alg 6%, HA 8%, CaCl_2_ 1M1h, st and (**d**,**e**) Alg 6%, HA 8%, CaCl_2_ 1M1h, GA, st. Aerogels were observed at two different magnifications.

scCO_2_ technology was employed as a biomaterial processing method, but it is also described as a sterilizing technique convenient for grafts prior to patient implantation [44], so this possibility was herein explored. The absence of microbial growth was found on TSA plates and TSB tubes after supercritical sterilization (Figure 9a). Textural and morphological properties of dual crosslinked aerogels were preserved after the sterilization procedure (Table 4, Figure 9b–e) with respect to the same aerogel formulations before this post-processing step (Table 2, Figure 3). Similar findings in terms of textural properties for other alginate composites sterilized with supercritical CO_2_ were previously reported [44]. Nevertheless, other polysaccharide sources for aerogels showed differing results when submitted to the same processing conditions [45]. These features highlight the importance of defining a sterilization strategy suitable for biomaterials components and structure. The mild conditions of temperature (40 °C), pressure (100 bar) and contact time (30 min) herein reported are therefore compatible with the sterilization of alginate-HA aerogels scaffolds without compromising their structural stability. These results provide future perspectives toward the development of implants in the regenerative medicine field with clinically appropriate sterility.

All formulations studied have acceptable textural properties and excellent biological behavior (cyto- and hemocompatibility) for BTE applications. Nevertheless, only the dual crosslinked scaffolds present the long-term stability as well as the structural integrity and bioactivity required to act as bone scaffolds. Regarding the HA concentration, aerogels manufactured with HA 8 wt.% present slightly better textural properties than other HA percentages while preserving their advanced biological performance and the printability required for the obtention of customized structures suitable for personalize medicine. All these features make this formulation a promising candidate to develop aerogel-based scaffolds for personalized BTE.

## 4. Conclusions

Osteoinductive alginate-HA aerogel scaffolds were successfully fabricated with a precise, customized, and dual porous structure via the combination of 3D-printing and aerogel technologies. An optimized scaffold formulation obtained by dual crosslinking with CaCl_2_ and GA provided long-term stability in an aqueous medium and the required bioactivity for promoting bone regeneration. The GA vapor crosslinking resulted in an effective method that can be used to obtain the textural properties and structural integrity required for customized BTE applications. Additionally, GA did not have any toxic effect or negative impact on the cell viability of fibroblasts and showed a high hemocompatibility for all the aerogels formulations tested, thus avoiding the detoxifying strategies commonly employed after this crosslinking strategy. Finally, aerogels under sterile conditions were successfully obtained by supercritical CO_2_ processing at mild conditions without compromising the structural integrity of dual crosslinked scaffolds. The high cytocompatibility, the suitable structure (pore size and porosity), the tunable macroscopic pattern obtained by 3D-printing and the sterile condition result in dual crosslinked aerogel scaffolds that represent a promising alternative for personalized BTE applications.

## Figures and Tables

**Table 1:** Dual crosslinked alginate-HA aerogel formulations studied.

Aerogel Formulations	Ink Composition		Crosslinking Strategy	
	Alginate (wt.%)	HA (wt.%)	Ionic Ageing	GA Vapor
Alg 6%, HA 0%, CaCl_2_ 1M1h	6	0	CaCl_2_ 1 M bath for 1 h	No
Alg 6%, HA 8%, CaCl_2_ 1M1h	6	8	CaCl_2_ 1 M bath for 1 h	No
Alg 6%, HA 16%, CaCl_2_ 1M1h	6	16	CaCl_2_ 1 M bath for 1 h	No
Alg 6%, HA 24%, CaCl_2_ 1M1h	6	24	CaCl_2_ 1 M bath for 1 h	No
Alg 6%, HA 0%, CaCl_2_ 1M1h, GA	6	0	CaCl_2_ 1 M bath for 1 h	Yes, 1 h
Alg 6%, HA 8%, CaCl_2_ 1M1h, GA	6	8	CaCl_2_ 1 M bath for 1 h	Yes, 1 h
Alg 6%, HA 16%, CaCl_2_ 1M1h, GA	6	16	CaCl_2_ 1 M bath for 1 h	Yes, 1 h
Alg 6%, HA 24%, CaCl_2_ 1M1h, GA	6	24	CaCl_2_ 1 M bath for 1 h	Yes, 1 h

**Table 2:** Textural properties of aerogel-based scaffolds obtained from aqueous 6 wt.% alginate inks with different HA concentrations (0, 8, 16, 24 wt.%) and crosslinked by different strategies: CaCl_2_ 0.5M1h/1M1h (aged aerogels), CaCl_2_ 1 M (non-aged aerogels) and GA (GA-crosslinked aerogels). Notation: A_BET_: specific BET surface area, d_p_: BJH-mean pore diameter, V_p_: BJH-specific pore volume.

Aerogel Scaffold	A_BET_ (m^2^/g)	d_p_ (nm)	V_p_ (cm^3^/g)	*ρ*_env_ (g/cm^3^)	*ρ*_skel_ (g/cm^3^)	*ε* (%)
Alg 6%, HA 0%, CaCl_2_ 1 M *	183 ± 9	19 ± 1	1.16 ± 0.06	0.14 ± 0.01 ^ǂ^	1.18 ± 0.16	86.58 ± 2.26
Alg 6%, HA 8%, CaCl_2_ 1 M *	118 ± 6	24 ± 1	0.99 ± 0.05	0.24 ± 0.03 ^ǂ^	1.72 ± 0.09	85.25 ± 4.02
Alg 6%, HA 16%, CaCl_2_ 1 M *	67 ± 3	26 ± 1	0.60 ± 0.03	0.29 ± 0.04 ^ǂ^	1.93 ± 0.04	79.78 ± 3.92
Alg 6%, HA 24%, CaCl_2_ 1 M *	29 ± 2	31 ± 2	0.21 ± 0.01	0.34 ±0.02 ^ǂ^	1.72 ± 0.14	76.68 ± 3.54
Alg 6%, HA 0%, CaCl_2_ 0.5M1h	453 ± 23	25 ± 1	3.21 ± 0.16	0.18 ± 0.01 ^ǂ^	1.81 ± 0.08	90.32 ± 0.81
Alg 6%, HA 0%, CaCl_2_ 1M1h	244 ± 12	24 ± 1	1.74 ± 0.1	0.19 ± 0.003	1.44 ± 0.09	86.78 ± 0.88
Alg 6%, HA 8%, CaCl_2_ 1M1h	81 ± 4	26 ± 1	0.68 ± 0.03	0.41 ± 0.003	2.29 ± 0.03	82.27 ± 0.24
Alg 6%, HA 16%, CaCl_2_ 1M1h	49 ± 3	28 ± 1	0.39 ± 0.02	0.55 ± 0.003	2.38 ± 0.04	76.86 ± 0.35
Alg 6%, HA 24%, CaCl_2_ 1M1h	65 ± 3	28 ± 1	0.69 ± 0.03	0.63 ± 0.003	2.71 ± 0.05	76.77 ± 0.47
Alg 6%, CaCl_2_ 1 M, GA	112 ± 6	19 ± 1	0.72 ± 0.04	0.22 ± 0.07 ^ǂ^	1.49 ± 0.05	85.45 ± 4.60
Alg 6%, HA 8%, CaCl_2_ 1 M, GA	91 ± 5	21 ± 1	0.66 ± 0.03	0.41 ± 0.09 ^ǂ^	1.84 ± 0.04	77.50 ± 4.80
Alg 6%, HA 0%, CaCl_2_ 1M1h, GA	26 ± 1	19 ± 1	0.15 ± 0.01	0.52 ± 0.09	1.96 ± 0.19	73.32 ± 2.62
Alg 6%, HA 8%, CaCl_2_ 1M1h, GA	28 ± 1	24 ± 1	0.21 ± 0.01	0.68 ± 0.09	2.16 ± 0.09	68.58 ± 1.41
Alg 6%, HA 16%, CaCl_2_ 1M1h, GA	21 ± 1	32 ± 2	0.24 ± 0.01	0.94 ± 0.20	2.33 ± 0.09	59.58 ± 1.47
Alg 6%, HA 24%, CaCl_2_ 1M1h, GA	21 ± 1	24 ± 1	0.19 ±0.01	0.97 ± 0.13	2.37 ± 0.05	59.14 ± 0.87

* Data obtained from the literature (ref. [7]).^ǂ^ Data calculated following the procedure reported in ref. [7]).

**Table 3:** Hemolytic activity tests for aerogel scaffolds. Positive control: Triton solution + blood; negative control: PBS solution pH 7.4 + blood.

Aerogel Scaffolds	Hemolytic Activity
Negative control	0.0 ± 2.0
Positive control	100.0 ± 0.2
Alg 6%, HA 24%, CaCl_2_ 1M1h	−1.3 ± 2.0
Alg 6%, HA 24%, CaCl_2_ 1M1h, GA	−1.8 ± 3.0

**Table 4:** Textural properties of sterile aerogel-based scaffolds obtained from aqueous 6 wt.% alginate inks with different HA contents (0, 8, 16, 24 wt.%) and crosslinked by ageing with CaCl_2_ 1M1h and by GA. Notation: A_BET_: specific BET surface area, d_p_: BJH-mean pore diameter, V_p_: BJH-specific pore volume.

Sterile Aerogel Scaffold	A_BET_ (m^2^/g)	d_p_ (nm)	V_p_ (cm^3^/g)
Alg 6%, HA 8%, CaCl_2_ 1M1h, st	105 ± 5	28 ± 1	0.89 ± 0.04
Alg 6%, HA 16%, CaCl_2_ 1M1h, st	68 ± 3	27 ± 1	0.52 ± 0.03
Alg 6%, HA 24%, CaCl_2_ 1M1h, st	56 ± 3	26 ± 1	0.41 ± 0.02
Alg 6%, HA 8%, CaCl_2_ 1M1h, GA, st	59 ± 3	25 ± 1	0.43 ± 0.02
Alg 6%, HA 16%, CaCl_2_ 1M1h, GA, st	30 ± 2	29 ± 1	0.27 ± 0.01
Alg 6%, HA 24%, CaCl_2_ 1M1h, GA, st	32 ± 2	23 ± 1	0.17 ± 0.01
